# Emergency Departments Need to Train and Hire More Sub‐Specialists in Addiction Medicine

**DOI:** 10.1002/aet2.70184

**Published:** 2026-05-11

**Authors:** Anthony Spadaro, Timothy Kelly, Jason Morris, Megan E. Heeney

**Affiliations:** ^1^ Department of Emergency Medicine University of Pennsylvania Philadelphia Pennsylvania USA; ^2^ Department of Emergency Medicine Icahn School of Medicine at Mount Sinai New York City New York USA; ^3^ Department of Emergency Medicine Highland Hospital Alameda Health System Oakland California USA

According to the 2024 National Survey on Drug Use and Health, 48.4 million people in the United States had a substance use disorder (SUD), while only 19.3% of patients with SUD received substance use treatment in the past year [1]. There are significant shortages in the addiction medicine workforce currently being addressed on systemic levels by increasing fellowship training programs and training medical professionals in the emergency departments (ED) and primary care to initiate medications for addiction treatment (MAT) [2]. Visits for substance use represent approximately 9.4% of ED presentations in the United States [3]. While many treatments for SUD have a robust evidence base, their uptake in the ED is limited. In one study, less than 10% of patients with opioid use disorder (OUD) were initiated on buprenorphine, and in another study only 7.4% of patients received take‐home naloxone after a nonfatal opioid overdose [4, 5]. There is a large gap in access to treatment, particularly for patients already suffering from healthcare disparities, and we have opportunities to bridge this gap and provide low barrier treatment and linkage to care through the ED [6]. Improving care for patients with SUDs in the ED will require increased training of emergency medicine providers on addiction‐related topics as well as EDs hiring more board‐certified addiction medicine physicians.

Leaders in graduate medical education have been calling for more addiction medicine education in residency across all specialties [7]. The importance of addiction medicine training within emergency medicine training has been noted by the Accreditation Council for Graduate Medical Education (ACGME) as proposed changes for 2027 include additional training in addiction medicine. This is echoing the growing understanding that trainees need to have the skills to initiate MAT and increase critical linkage to care for vulnerable patients. While didactic lectures can provide an important foundation for understanding SUDs, trainees should have real clinical experience with physicians with expertise in addiction medicine. Emergency medicine residencies should provide clinical experiences dedicated to the care of patients with SUD, whether that be in a hospital‐based addiction medicine consult service, detox center, bridge clinic, or street medicine. While many EDs provide extensive experience caring for patients with SUD as part of the day‐to‐day clinical experience of working in an ED, dedicated time to learning about more advanced clinical addiction medicine will improve the breadth and depth of knowledge emergency medicine residents will have on addiction medicine topics.

While we all pursued addiction medicine fellowships to become experts in the care of patients with SUD in the ED, we used elective time during our emergency medicine residencies to gain additional clinical experience and expertise as residents. Having an addiction medicine experience as an ACGME requirement will be vital to bring the emergency medicine workforce up to speed with the advancements in addiction care that have taken place and expose new generations of trainees to a field that they can pursue fellowship in. Looking to other specialties, in one internal medicine residency, providing enhanced SUD curriculum facilitated residents feeling more prepared to diagnose and treat SUD [8]. Currently, there are 285 emergency medicine residencies and only 109 addiction medicine fellowships; many residencies do not have affiliations with established addiction medicine fellowships to provide clinical addiction experiences. Recruiting faculty with board certification in addiction medicine should be a top priority for EDs that want to provide the most thorough addiction medicine education for their residencies and provide the highest quality clinical care for their patients with SUDs.

Hiring more faculty in the ED who are addiction medicine boarded will embed local champions in a department who can bring novel innovations from addiction medicine research into the ED clinical environment. For example, there is growing interest in providing long‐acting injectable buprenorphine to patients with OUD from the ED, and having ED faculty with addiction medicine expertise will be necessary to implement uptake of this practice as successful practice change often requires multicomponent interventions [9]. ED faculty with board certification in addiction medicine can also help EDs uptake society guideline endorsed practices such as starting naltrexone for AUD, which is currently underutilized from the ED.

In addition to hiring faculty with board certification in addiction medicine, EDs should increase the number of addiction‐related services that they offer. In particular, ED‐based peer recovery services are valuable resources for patients and providers as they help connect patients with outpatient care, enhance motivation for behavioral change, increase uptake of medications for addiction treatment, and reduce stigma in EDs [10]. In addition to improving patient care and patients' experience in the ED, peer recovery services have been found to provide cost savings to the health care system. Emergency medicine physicians trained in addiction medicine are often instrumental in both bringing these types of evidence‐based, cost‐saving services into the ED and in guiding their successful implementation.

Anecdotally we have all taken faculty positions at institutions that are enthusiastically investing in addiction medicine and hope that other institutions follow suit. Our jobs include clinical work in the ED, running a bedside hospital‐based addiction consult service, working in a bridge clinic, research, and administrative tasks. Although it may seem difficult to integrate work outside of the ED with the 24/7 nature of ED shifts, we have found that it is possible to smoothly switch between divisions due to the discrete time‐bound nature of some of this work. Additionally, we have all benefited from having chairs in our EDs that value the importance of our addiction medicine work and compensate for our time doing clinical addiction medicine work similar to that of our time in the ED. Having schedulers from both the ED and addiction medicine division who are understanding of the complexities of our schedules has been valuable. Not only is this mix of clinical duties and sites feasible with supportive leadership and administrative staff, it is contributing to our job satisfaction and will undoubtedly lead to improved retention and less burnout, something the field of emergency medicine is particularly prone to. Emergency medicine physicians who are board certified in addiction medicine or considering fellowship and looking for jobs should advocate for themselves by highlighting what their expertise can bring to the department. In addition to advancing addiction medicine education in the department, developing clinical services, and innovating practice, emergency medicine physicians who are board certified in addiction medicine can use their expertise to develop research projects that bring in valuable research grants into departments. As the drug supply is constantly changing, and there remain many areas of improvement in addressing the overdose epidemic, ED‐based addiction medicine research is all the more important to figure out ways of improving care for our patients with SUD.

Developing an emergency medicine workforce to address the current overdose epidemic and innovating ED care of patients with SUDs will take time. From our recent experience completing fellowships in addiction medicine, we can affirm that there is enthusiasm among emergency medicine trainees to gain the skills and expertise involved in subspecialty training in addiction medicine. Departments of Emergency Medicine should foster this enthusiasm by promoting addiction medicine curriculum in their residencies and hiring faculty with board certification in emergency medicine. A rising tide raises all boats, and supporting board‐certified addiction medicine physicians within EDs will improve the care of patients with SUDs across the entire department.

## Author Contributions

**Anthony Spadaro:** conceptualization, writing – review and editing, supervision, writing – original draft. **Timothy Kelly:** conceptualization, writing – original draft, writing – review and editing, supervision. **Jason Morris:** conceptualization, writing – original draft, writing – review and editing, supervision. **Megan E. Heeney:** conceptualization, writing – original draft, writing – review and editing, supervision.

## Funding

Dr. Spadaro, Kelly, Morris, Heeney was supported by R25DA033211 from the National Institute on Drug Abuse.

## Disclosure

A.S., T.K., J.M., M.H. reports grant support by R25DA033211 from the National Institute on Drug Abuse to support research conceived of and conducted by A.S., T.K., J.M., and M.H.

## Conflicts of Interest

The authors declare no conflicts of interest.

## Data Availability

Data sharing not applicable to this article as no datasets were generated or analysed during the current study.
